# A hybrid biological neural network model for solving problems in cognitive planning

**DOI:** 10.1038/s41598-022-11567-0

**Published:** 2022-06-23

**Authors:** Henry Powell, Mathias Winkel, Alexander V. Hopp, Helmut Linde

**Affiliations:** 1 Merck KGaA, Darmstadt, Germany; 2 University of Glasgow, Glasgow, Scotland UK; 3 Transylvanian Institute of Neuroscience, Cluj-Napoca, Romania

**Keywords:** Computational biology and bioinformatics, Neuroscience, Systems biology

## Abstract

A variety of behaviors, like spatial navigation or bodily motion, can be formulated as graph traversal problems through cognitive maps. We present a neural network model which can solve such tasks and is compatible with a broad range of empirical findings about the mammalian neocortex and hippocampus. The neurons and synaptic connections in the model represent structures that can result from self-organization into a cognitive map via Hebbian learning, i.e. into a graph in which each neuron represents a point of some abstract task-relevant manifold and the recurrent connections encode a distance metric on the manifold. Graph traversal problems are solved by wave-like activation patterns which travel through the recurrent network and guide a localized peak of activity onto a path from some starting position to a target state.

## Introduction

Building a bridge between structure and function of neural networks is an ambition at the heart of neuroscience. Historically, the first models studied were simplistic artificial neurons arranged in a feed-forward architecture. Such models are still widely applied today—forming the conceptual basis for Deep Learning. They have shaped our intuition of neurons as “feature detectors” which fire when a certain approximate configuration of input signals is present, and which aggregate simple features to more and more complex ones layer by layer. Yet in the brain, the vast majority of neural connections is recurrent, and although several possible explanations of their function have been proposed^1–3^, their computational purpose is still little understood^4^.

In the present paper, we propose a new algorithmic role which recurrent neural connections might play, namely as a computational substrate to solve graph traversal problems. We argue that many cognitive tasks like navigation or motion planning can be framed as finding a path from a starting position to some target position in a space of possible states. The possible states may be encoded by neurons via their “feature-detector property”. Allowed transitions between nearby states would then be encoded in recurrent connections, which can form naturally via Hebbian learning since the feature detectors’ receptive fields overlap. They may eventually form a “map” of some external system. Activation propagating through the network can then be used to find a short path through this map. In effect, the neural dynamics then implement an algorithm similar to Breadth-First Search on a graph.

## Proposed model

### A network of neurons that represents a manifold of stimuli

**Figure 1:**
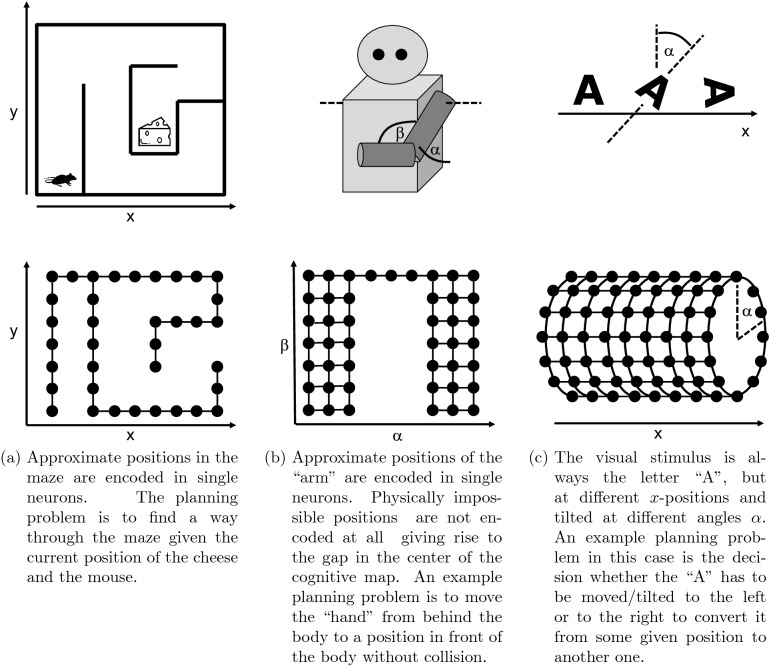
Three examples of stimuli-generating processes and recurrent neural networks representing the corresponding manifold of stimuli. (**a**) Approximate positions in the maze are encoded in single neurons. The planning problem is to find a way through the maze given the current position of the cheese and the mouse. (**b**) Approximate positions of the "arm" are encoded in single neurons. Physically impossible positions are not encoded at all giving rise to the gap in the center of the cognitive map. An example planning problem is to move the "hand" from behind the body to a position in front of the body without collision. (**c**) The visual stimulus is always the letter "A", but at different x-positions and tilted at different angles *α*. An example planning problem in this case is the decision whether the "A" has to be moved/tilted to the left or to the right to convert it from some given position to another one.

We consider a neural network which is exposed to some external stimuli-generating process under the assumption that the possible stimuli can be organized in some continuous manifold in the sense that similar stimuli are located close to each other on this manifold. For example, in the case of a mouse running through a maze, all possible perceptions can be associated with a particular position in a two-dimensional map, and neighboring positions will generate similar perceptions, see Fig. 1a.

Proprioception, i. e. the sense of location of body parts, can also be a source of stimuli. For example, for a simplified arm with two degrees of freedom every possible position of the arm corresponds to one specific stimulus, cf. Fig. 1b. All possible stimuli combined give rise to a two-dimensional manifold. The example also shows that the manifold will usually be restricted since not every conceivable combination of two joint angles might be a physically viable position for the arm.

The manifold of potential stimuli needs not necessarily be embedded in a flat Euclidean space as in the case of the maze. For example, if the stimuli are two-dimensional figures which can be shifted horizontally or rotated on a screen, the corresponding manifold is two-dimensional (one translational parameter plus one for the rotation angle) but it is not isomorphic to a flat plane since a change of the rotation angle by $2\pi$ maps the figure onto itself again, see Fig. 1c.

We assume that such manifolds of stimuli are approximated by the connectivity structure of a neural network which forms via a learning process. The result is a neural structure which we call a *cognitive map*. The defining property of a cognitive map is that is has a neural encoding for every possible stimulus and that two similar stimuli, i. e. stimuli which are close to each other in the manifold of stimuli, are represented by similar encodings, i. e. encodings which are close to each other in the cognitive map (of course, we do not imply that two neurons which are close to each other in the connectivity structure are also close to each other with respect to their physical location in the neural tissue).

**Figure 2:**
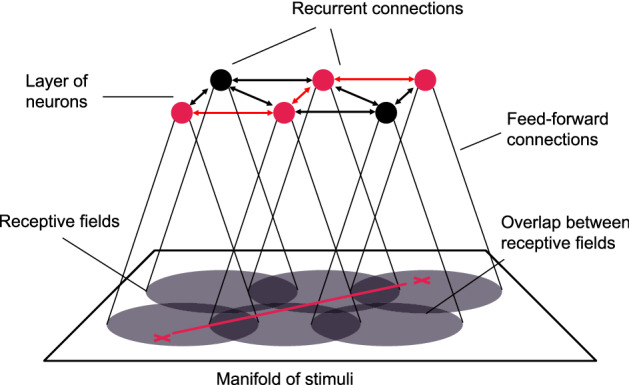
In the model, the recurrent connections within a single layer of neurons approximate the topology of the manifold of stimuli. During the learning process, the strongest recurrent connections are formed between neurons with overlapping receptive fields. The problem of finding a route through the manifold (red line) is thus approximated by the problem of finding a path through the graph of recurrent neural connections (red path).

For the model, we make a very simplistic choice and assume a single-neuron encoding, i. e. the manifold of stimuli is covered by the receptive fields of individual neurons. Each such receptive field is a small localized area in the manifold and two neighboring receptive fields may overlap, see Fig. 2. Such an encoding is a typical outcome for a single layer of neurons which are trained in a competitive Hebbian learning process^5^.

The key idea of the model is that solving a problem that can be formulated as a planning problem in the manifold of stimuli, can be solved as a planning problem in a corresponding cognitive map. To this end, it is not enough to consider the cognitive map as a set of individual points, but its topology must be known as well. This topological information will be encoded in the recurrent connections of the neural network.

It seems natural that a neural network could learn this topology via Hebbian learning: Two neurons with close-by receptive fields in the manifold will be excited simultaneously relatively often because their receptive fields overlap. Consequently, recurrent connections within the cognitive map will be strengthened between such neurons and the topology of the neural network will approximate the topology of the manifold, see Fig. 2. This idea has been explored in more detail by Curto and Itskov in^6^. Indeed, previous work on the formation of neocortical maps that code for ocular dominance and stimulus orientation suggest that the formation of cognitive maps could well occur in this fashion^7^. For a review and comparison of these kinds of cognitive maps see^8^. Recent studies also show that recurrent neural networks might serve even more purposes, for example for working memory^9,10^ or image recognition^11^.

To avoid confusion with related concepts in machine learning, note that the present definition of recurrence is not exactly the same as the one used, for example, in Long Short-Term Memory networks^12^. Those algorithms employ recurrent connections as a loop to mix some input signal of a neural network with the output signal from a previous time step. The present model, however, separates between the primary excitation by some external stimulus via feed-forward connections and the resulting dynamics of the network mediated by the recurrent connections as described in the following.

### Dynamics required for solving planning problems

Having set up a network that represents a manifold of stimuli, we need to endow this network of feed-forward and recurrent connections with dynamics. We do so by imposing two interacting mechanisms.

First, the neurons in the network should exhibit continuous attractor dynamics^13^: If a “clique” of a few tightly connected neurons are activated by a stimulus via the corresponding feed-forward pass, they keep activating each other while inhibiting their wider neighborhood. The result is a self-sustained, localized neural activity surrounded by a “trench of inhibition”. In the model, this encodes the as-is situation or the starting position for the planning problem. Such a state is called an “attractor” since it is stable under small perturbations of the dynamics, and it is part of a continuous landscape of attractors with different locations across the network. The dynamics of these kinds of bumps of activity in neural sheets of different kinds has been studied in depth in^14^ and applied to more general problems in neurosciene^15^ but have not, as of yet, been used as means to solve planning problems in the way proposed here.

Second, the neural network should allow for wave-like expansion of activity. If a small number of close-by neurons are activated by some hypothetical executive brain function (i. e. not via the feed-forward pass), they activate their neighbors, which in turn activate theirs, and so on. The result is a wave-like front of activity propagating through the recurrent network. The neurons which have been activated first encode the to-be state or the end position of the planning problem.

**Figure 3:**
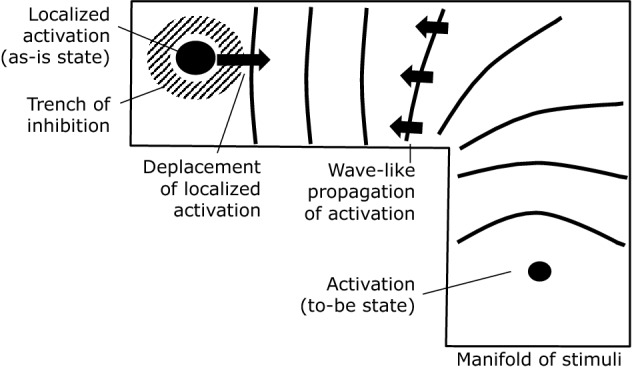
The as-is state of the system is encoded in a stable, localized, and self-sustained peak of activity surrounded by a “trench” of inhibition (top left corner). A planning process is started by stimulating the neurons which encode the to-be position (bottom right corner). The resulting waves of activity travel through the network and interact with the localized peak. Each incoming wave front shifts the peak slightly towards its direction of origin. Note that, for reasons of simplicity, we did not draw the neural network in this figure but only the manifold which it approximates.

The key to solving a planning problem is in the interaction between the two types of dynamics, namely in what happens when the expanding wave front hits the stationary peak of activity. On the side where the wave is approaching it, the “trench of inhibition” surrounding the peak is in part neutralized by the additional excitatory activation from the wave. Consequently, the containment of the activity peak is somewhat “softer” on the side where the wave hit it and it may move a step towards the direction of the incoming wave. This process repeats, leading to a small change of position with every incoming wave front. The localized peak of excitation will follow the wave fronts back to their source, thus moving along a route through the manifold from start to end position, see Fig. 3.

The two types of dynamics described above are seemingly contradictory, since the first one restricts the system to localized activity, while the second one permits a wave-like propagation of activity throughout the system. To resolve the conflict in numerical simulations, we have split the dynamics into a *continuous attractor layer* and a *wave propagation layer*, which are responsible for different aspects of the system’s dynamical behaviour. We discuss the concepts of a numerical implementation in the section “Implementation in a numerical proof-of-concept” and ideas for a biologically more plausible implementation in the “Discussion” section.

### Connection to real-life cognitive processes

To make the proposed concept more tangible, we present a rough sketch of how it could be embedded in a real-life cognitive process along with a speculative proposal for its anatomical implementation in the special case of motor control.

As an example, we consider a human grabbing a cup of coffee and we explain how the presented model complements and details the processes described in^16^ for that particular case. According to our hypothesis, the as-is position of the subject’s arm is encoded as a localized peak of activity in the cognitive map encoding the complex manifold of arm positions. Anatomically, this cognitive map is certainly of a more complicated structure than the one in our simple model and it is possibly shared between primary motor cortex and primary somatosensory cortex.

We assume that the encoding of the arm’s state works in a bi-directional way, somewhat like the string of a puppet: When the arm is moved by external forces, the neural representation of its position mediated by afferent somatosensory signals moves along with it. On the other hand, if the representation in the cortical map is changed slightly by some cognitive process, then some hypothetical control mechanism of the primary motor cortex sends efferent signals to the muscles in an attempt to make the arm follow its neural representation and bring the limb and its representation back into congruence.

If now the human subject decides to grab the cup of coffee, some executive brain function with heavy involvement from prefrontal cortex constructs a to-be state of holding the cup: The final position of the hand with the fingers around the cup handle is what the person consciously thinks of. The high-level instructions generated by prefrontal cortex are possibly translated by the premotor cortex into a specific target state in the cognitive map that represents the manifold of possible arm positions. The neurons of the primary motor cortex and/or the primary somatosensory cortex representing this target state are thus activated.

The activation creates waves of activity propagating through the network, reaching the representation of the as-is state and shifting it slightly towards the to-be state. The hypothetical muscle control mechanism reacts on this disturbance and performs a motor action to keep the physical position of the arm and its representation in the cognitive map in line. As long as the person implicitly represents the to-be state, the arm “automatically” performs the complicated sequence of many individual joint movements which is necessary to grab the cup.

This concept can be extended to flexibly consider restrictions that have not been hard-coded in the cognitive map by learning. For example, in order to grab the cup of coffee, the arm may need to avoid obstacles on the way. To this end, the hypothetical executive brain function which defines the target state of the hand could also temporarily “block” certain regions of the cognitive map (e. g. via inhibition) which it associates with the discomfort of a collision. Those parts of the network which are blocked cannot conduct the “planning waves” anymore and thus a path around those regions will be found.

### Implementation in a numerical proof-of-concept

To substantiate the presented conceptual ideas, we performed numerical experiments using multiple different setups. In each case, the implementation of the model employs two neural networks that both represent the same manifold of stimuli.

The continuous attractor layer is a sheet of neurons that models the functionality of a network of place cells in the human hippocampus^17,18^. Each neuron is implemented as a rate-coded cell embedded in its neighborhood via short-range excitatory and long-range inhibitory connections as in^19^. This structure allows the formation of a self-sustaining “bump” of activity, which can be shifted through the network by external perturbations.

The wave propagation layer is constructed with an identical number of excitatory and inhibitory Izhikevich neurons^20,21^, properly connected to allow for stable signal propagation across the manifold of stimuli. The target node is permanently stimulated, causing it to emit waves of activation which travel through the network.

The interaction between the two layers is modeled in a rather simplistic way. As in^19^, a time-dependent direction vector was introduced in the synaptic weight matrix of the continuous attractor layer. It has the effect of shifting the synaptic weights in a particular direction which in turn causes the location of the activation bump in the attractor layer to shift to a neighbouring neuron. The direction vector is updated whenever a wave of activity in the wave propagation layer newly enters the region which corresponds to the bump in the continuous attractor layer. Its direction is set to point from the center of the bump to the center of the overlap area between bump and wave, thus causing a shift of the bump towards the incoming wave fronts.

For more details on the implementation, see “Methods and experiments” below.

### Results of the numerical experiments

**Figure 4:**
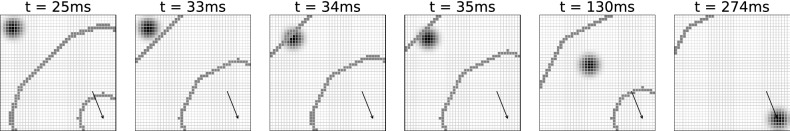
Activity in the wave propagation layer (greyish lines) and the continuous attractor layer (circular blob-like structure) overlaid on top of each other at different time points during the simulation. The grid signifies the neural network structure, i. e. every grid cell in the visualization corresponds to one neuron in each, the wave propagation layer and the continuous attractor layer. The position of the external wave propagation layer stimulation (to-be state) is shown with an arrow. Starting from an initial position in the top left of the sheet, the activation bump traces back the incoming waves to their source in the bottom right.

In a very simple initial configuration, the path finding algorithm was tested on a fully populated quadratic grid of neurons as described before. Figure 4 shows snapshots of wave activity and continuous attractor position at some representative time points during the simulation. As expected, stimulation of the wave propagation layer in the lower right of the cognitive map causes the emission of waves, which in turn shift the bump in the continuous attractor layer from its starting position in the upper left towards its target state.

**Figure 5:**
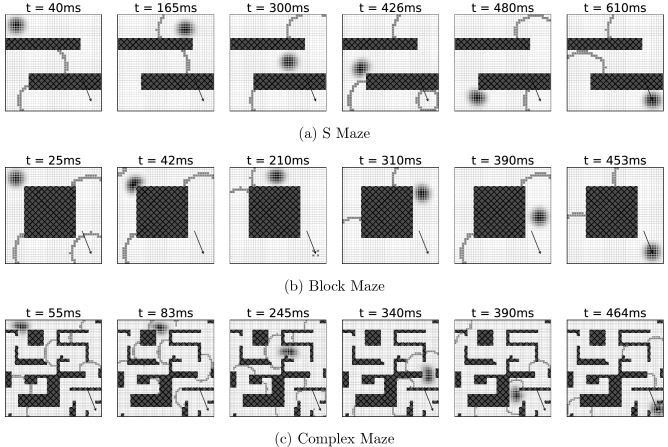
Simulations where specific portions of the neural layers were blocked for traversal (dark hatched regions) show the model’s capability of solving complex planning problems. Note, that especially in the very fine structure of Fig. 5c leftover excitation can trigger waves apparently spontaneously in the simulation region, such as at the right center at $t={83}\,\hbox {ms}$. As the corresponding neurons are not constantly stimulated, these are usually singular events that do not disturb the overall process (Supplementary Videos).

As described in the section “Connection to real-life cognitive processes” above, the manifold of stimuli represented by the neural network can be curved, branched, or of different topology, either permanently or temporarily. The purpose of the model is to allow for a reliable solution to the underlying graph traversal problems independent of potential obstacles in the networks. For this reason we investigated whether the bump of activation in the continuous attractor layer was able to successfully navigate through the graph from the starting node to the end node in the presence of nodes that could not be traversed. To test this idea we constructed different “mazes”, blocking off sections of the graph by zeroing the synaptic connections of the respective neurons in the wave propagation layer and by clamping activation functions of the corresponding neurons in the continuous attractor layer to zero, see Fig. 5. We found that in all these setups, the algorithm was able to successfully navigate the bump in the continuous attractor layer through the mazes.

### Relation to existing graph traversal algorithms

To conclude this section, we highlight a few parallels between the presented approach and the classical Breadth-First Search (*BFS*) algorithm.

*BFS*  begins at some start node $s$ of the graph and marks this node as “visited”. In each step, it then chooses one node which is “visited” but not “finished” and checks whether there are still unvisited nodes that have an edge to this node. If so, the corresponding nodes are also marked as “visited”, the current node is marked as “finished” and another iteration of the algorithm is started.

The approach presented here is a *parallelized* variant of this algorithm. Assuming that all neurons always obtain sufficient current to become activated, the propagating wave corresponds to the step of the algorithm in which the neighbors of the currently considered node are investigated. In contrast to *BFS*, the algorithm performs this step for all candidate nodes in a single step. That is, it considers *all* nodes currently marked as visited, checks the neighbors of all these nodes *at once* and marks them as visited if necessary.

Having all ingredients of the proposed conceptual framework in place, the following section reviews some experimental evidence indicating that it could in principle be employed by biological brains.

## Empirical evidence

### Cognitive maps

The concept of “cognitive maps” was first proposed by Edward Tolman^22,23^, who conducted experiments to understand how rats were able to navigate mazes to seek rewards.

A body of evidence suggests that neural structures in the hippocampus and enthorinal cortex potentially support cognitive maps used for spatial navigation^17,24,25^. Within these networks, specific kinds of neurons are thought to be responsible for the representation of particular aspects of cognitive maps. Some examples are place cells^17,24^ which code for the current location of a subject in space, grid cells which contribute to the problem of locating the subject in that space^26^ as well as supporting the stabilisation of the attractor dynamics of the place cell network^19^, head-direction cells^27^ which code for the direction in which the subject’s head is currently facing, and reward cells^28^ which code for the location of a reward in the same environment.

The brain regions supporting spatially aligned cognitive maps might also be utilized in the representation of cognitive maps in non-spatial domains: In^29^, fMRI recordings taken from participants while they performed a navigation task in a non-spatial domain showed that similar regions of the brain were active for this task as for the task outlined in^30^ where participants navigated a virtual space using a VR apparatus. Further, according to^31^, activation of neurons in the hippocampus (one of the principal sites for place cells) is indicative of how well participants were able to perform in a task related to pairing words. Supporting this observation with respect to the role played by these brain regions in the operation of abstract cognitive maps^32^, found that lesions to the hippocampus significantly impaired performance on a task of associating pairs of odors by how similar they smelled. Finally, complementing these findings, rat studies have shown that hippocampal cells can code for components in navigation tasks in auditory^33,34^, olfactory^35^, and visual^36^ task spaces.

### Feed-forward and recurrent connections

As described in the section “A network of neurons that represents a manifold of stimuli”, the proposed model is built around a particular *theme of connectivity*: Each neuron represents a certain pattern in sensory perception mediated via feed-forward connections. In addition, recurrent connections between two neurons strengthen whenever they are activated simultaneously. In the following, we give an overview of some relevant experimental observations which are consistent with this mode of connectivity.

The most prominent example of neurons which are often interpreted as pattern detectors are the cells in primary visual cortex. These neurons fire when a certain pattern is perceived at a particular position and orientation in the visual field. On the one hand, these neurons receive their feed-forward input from the lateral geniculate nucleus. On the other hand, they are connected to each other through a tight network of recurrent connections. Several studies (see e. g.^37–39^) have shown that two such cells are preferentially connected when their receptive fields are co-oriented and co-axially aligned. Due to the statistical properties of natural images, where elongated edges appear frequently, such two cells can also be expected to be positively correlated in their firing due to feed-forward activation.

The somatosensory cortex is another brain region where several empirical findings are in line with the postulated theme of connectivity. Experiments on non-human primates suggest that “3b neurons act as local spatiotemporal filters that are maximally excited by the presence of particular stimulus features”^40^.

Regarding the recurrent connections in somatosensory cortex, some empirical support stems from the well-studied rodent barrel cortex. Here, the animal’s facial whiskers are represented somatotopically by the columns of primary somatosensory cortex. Neighboring columns of the barrel cortex are connected via a dense network of recurrent connections. Sensory deprivation studies indicate that the formation of these connections depends on the feed-forward activation of the respective columns: If the whiskers corresponding to one of the columns are trimmed during early post-natal development, the density of recurrent connections with this column is reduced^41,42^. Conversely, synchronous co-activation over the course of a few hours can lead to increased functional connectivity in the primary somatosensory cortex^43^.

The primary somatosensory cortex also receives proprioceptive signals from the body which represent individual joint angles. Taken as a whole, these signals characterize the current posture of the animal and there is an obvious analogy to the arm example, cf. Fig. 1b. We are not aware of any experimental results regarding the recurrent connections between proprioception detectors, but it seems reasonable to expect that the results about processing of tactile input in the somatosensory cortex can be extrapolated to the case of proprioception. This would imply that a recurrent network structure roughly similar to Fig. 1b should emerge and thus support the model for controlling the arm.

Area 3a of the somatosensory cortex, whose neurons exhibit primarily proprioceptive responses, is also densely connected to the primary motor cortex. It contains many corticomotoneuronal cells which drive motoneurons of the hand in the spinal cord^44^. This tight integration between sensory processing and motor control might be a hint that the hypothetical string-of-a-puppet muscle control mechanism from the section on the “Connection to real-life cognitive processes” is not too far from reality.

In summary, evidence from primary sensory cortical areas seems to suggest a common cortical theme of connectivity in which neurons are tuned to specific patterns in their feed-forward input from other brain regions, while being connected intracortically based on statistical correlations between these patterns.

### Wave phenomena in neural tissue

There is a large amount of empirical evidence for different types of wave-like phenomena in neural tissue. We summarize some of the experimental findings, focusing on fast waves (a few tens of $\hbox {cm}\,\hbox {s}^{-1}$). These waves are suspected to have some unknown computational purpose in the brain^45^ and they seem to bear the most resemblance with the waves postulated in the model.

Using multielectrode local field potential recordings, voltage-sensitive dye, and multiunit measurements, traveling cortical waves have been observed in several brain areas, including motor cortex, visual cortex, and non-visual sensory cortices of different species. There is evidence for wave-like propagation of activity both in sub-threshold potentials and in the spatiotemporal firing patterns of spiking neurons^46^.

In the motor cortex of wake, behaving monkeys, Rubino et al.^47^ observed wave-like propagation of local field potentials. They found correlations between some properties of these wave patterns and the location of the visual target to be reached in the motor task. On the level of individual neurons, Takahasi et al. found a “spatiotemporal spike patterning that closely matches propagating wave activity as measured by LFPs in terms of both its spatial anisotropy and its transmission velocity”^48^.

In the visual cortex, a localized visual stimulus elicits traveling waves which traverse the field of vision. For example, Muller et al. have observed such waves rather directly in single-trial voltage-sensitive dye imaging data measured from awake, behaving monkeys^49^.

### Spatial navigation using place cells

Finding a short path through a maze-like environment, cf. Fig. 1a, is one of the planning problems the model is capable of solving. In this case, each neuron of the continuous attractor layer represents a “place cell” which encodes a particular location in the maze.

Place cells were discovered by John O’Keefe and Jonathan Dostrovsky in 1971 in the hippocampus of rats^17^. They are pyramidal cells that are active when an animal is located in a certain area (“place field”), of the environment. Place cells are thought to use a mixture of external sensory information and stabilizing internal dynamics to organize their activity: On the one hand, they integrate external environmental cues from different sensory modalities to anchor their activity to the real world. This is evidenced by the fact that their activity is affected by changes in the environment and that it is stable under a removal of a subset of cues^50,51^. On the other hand, firing patterns are then stabilized and maintained by internal network dynamics as cells remain active under conditions of total sensory deprivation^52^. Collectively, the place cells are thought to form a cognitive map of the animal’s environment.

### Targeted motion caused by localized neuron stimulation

In 2002, Graziano et al. reported results from electrical microstimulation experiments in the primary motor and premotor cortex of monkeys^53^. Stimulation of different sites in the cortical tissue for a duration of 500 ms resulted in complex body motions involving many individual muscle commands. The stimulation of one particular site typically led to smooth movements with a certain end state, independent of the initial posture of the monkey, while stimulating a different location in the cortical tissue led to a different end state. In terms of the model presented here, this would be explained by two wave fronts propagating in opposite directions away from the to-be location, only one of which hits the localized peak of activity encoding the as-is location and pulls it closer to the to-be state. Graziano et al. also reported that the motions stopped as soon as the electrical stimulus was turned off. This is fully consistent with our model, where stopping the to-be activation means that no more wave fronts are created and thus the as-is peak of activity remains where it is.

After this original discovery by Graziano et al. in 2002, several additional studies have confirmed and extended their results, see^54^ for an overview. The neural structures which cause the bodily motions towards a specific target state have been named *ethological maps* or *action maps*^54^.

Furthermore, several studies suggest that such action maps are shaped by experience: Restricting limb movements for thirty days in a rat can cause the action map to deteriorate. A recovery of the map is observed during the weeks after freeing the restrained limb^55^. Conversely, a reversible local deactivation of neural activity in the action map can temporarily disable a grasping action in rats^56^. A permanent lesion in the cortical tissue can disable an action permanently. The animal can re-learn the action, though, and the cortical tissue reorganizes to represent the newly re-learnt action at a different site^57^. These observed plasticity phenomena are fully in line with our model which emphasises a self-organized formation of the cognitive map via Hebbian processes both for the feature learning and for the construction of the recurrent connections.

### Participation of the primary sensory cortex in non-sensory tasks

For the first two examples in Fig. 1, the association with a planning task is obvious. Our third example, the geometric transformations of the letter “A”, may appear a bit more surprising, though: After all, the neural structures in visual sensory cortex would then be involved in “planning tasks”. The tissue of at least V1 fits the previously explained theme of connectivity, but it is often thought of as a pure perception mechanism which aggregates optical features in the field of vision and thus performs some kind of preprocessing for the higher cortical areas.

However, there is evidence that the visual sensory cortex plays a much more active role in cognition than pure feature detection on the incoming stream of visual sensory information. In particular, the visual cortex is active in visual imagery, that is, when a subject with closed eyes mentally imagines a visual stimulus^58^.

Based on such findings, it has been suggested that “the visual cortex is something akin to a ‘representational blackboard’ that can form representations from either the bottom-up or top-down inputs”^58^. In our model, we take this line of thinking one step further and speculate that the early visual cortex does not only represent visual features, but that it also encodes possible transformations like rotation, scaling or translation via its recurrent connections. In this view, the “blackboard” becomes more of a “magnetic board” on which mental images can be placed and shifted around according to rules which have been learned by experience.

Of course, despite the over-simplifying Fig. 1c, we do not intend to imply that there were any neurons in the visual cortex with a complex pattern like the whole letter “A” as a receptive field. In reality, we would expect the letter to be represented in early visual cortex as a spatio-temporal multi-neuron activity pattern. The current version of our model, on the other hand, allows for single-neuron encoding only and thus reserves one neuron for each possible position of the letter. We will discuss this and other limitations of the proposed model in the “Discussion” section.

### Temporal dynamics

The concept presented in this article implies predictions about the temporal dynamics of cognitive planning processes which can be compared to experiments: The bump of activity only starts moving when the first wave front arrives. Assuming that every wave front has a similar effect on the bump, its speed of movement should be proportional to the frequency with which waves are emitted. Thus both the time until movement onset and the duration of the whole planning process should be proportional to the length of the traversed path in the cortical map. Increased frequency of wave emission should accelerate the process.

One supporting piece of evidence is provided by mental imagery: Experiments in the 1970s^59,60^ have triggered a series of studies on mental rotation tasks, where the time to compare a rotated object with a template has often been found to increase proportionally with the angle of rotation required to align the two objects.

In the case of bodily motions, the total time to complete the cognitive task is not a well suited measure since it strongly depends on mechanical properties of the limbs. Yet for electrical stimulation of the motor cortex (cf. “Targeted motion caused by localized neuron stimulation” section) Graziano et al. report that the speed of evoked arm movements increases with stimulation frequency^61^. Assuming that this frequency determines the rate at which the hypothetical waves of activation are emitted, this is consistent with our model.

In addition, our model makes the specific prediction that the latency between stimulation and the onset of muscle activation should increase with the distance between initial and target posture. The reason is that the very first wave front needs to travel through the cognitive map before the bump of activation starts being shifted, and only then muscular activation can be triggered by the bump’s deflection. The travel time of this wave front thus becomes an additive component of the total latency and it can be expected to be roughly proportional to the distance between initial and target posture as measured in the metric of the cognitive map. We are not aware of any studies having examined this particular relationship yet.

## Discussion

The model proposed here is, to the best of our knowledge, the first model that allows for solving graph problems in a biological plausible way such that the solution (i. e. the specific path) can be calculated directly on the neural network as the only computational substrate.

Similar approaches and models have been investigated earlier, especially in the field of neuromorphic computing. For example, in^62–66^ graphs are modeled using neurons and synapses, and computations are performed by exciting specific neurons which induces propagation of current in the graph and observing the spiking behavior. Also, models using two or more cell layers and spiking neural neurons have been used for unsupervised learning of orientation, disparity, and motion representations^67^ or modeling the tactile processing pathway^68^. In addition, recurrent neural networks were recently also used to model and analyze working memory^9,10^ or image recognition tasks^11^. These models are however either designed for very specific tasks^68^, do not guarantee a stable performance^11^ or lack biological plausibility^9,10,67^ . Furthermore^69^, describes another neural computation mechanism which “might be a general computational mechanism of cortical circuits”^69^ using circuit models of spiking neurons. This mechanism is developed for understanding how spontaneous activity is involved in visual processing and is not investigated in terms of its applicability for solving planning problems.

Although some models are more general than the one presented here and allow for solving more complex problems like dynamic programs^63^, enumeration problems^65^ or the longest shortest path problem^66^, we are not aware of any model explicitly discussing the biological plausibility despite the need for more neurobiologically realistic models^70^. In fact, most of these approaches are far from being biologically plausible as they e. g. require additional artificial memory^63^ or a preprocessing step that changes the graph depending on the input data^66^. Also, the model of Muller et al.^62^ as well as the very recent model of Aimone et al.^64^ which are biologically more plausible do not discuss how a specific path can then be computed in the graph, even if the length of a path can be calculated^64^. In addition, some models try to describe actually observed wave propagation in the brain^71,72^.

In the following we discuss limitations of the presented model and potential avenues for further research.

### Single-neuron vs. multi-neuron encoding

In our model, each point on a cortical map is represented by a single neuron and a distance on the map is directly encoded in a synaptic strength between two neurons. The graph of synaptic connections can therefore be considered as a coarse-grained version of the underlying manifold of stimuli. Yet such a single-neuron representation is possible only for manifolds of a very low dimension, since the number of points necessary to represent the manifold grows exponentially with each additional dimension. For tasks like bodily movement, where dozens of joints need to be coordinated, the number of neurons required to represent every possible posture in a single-neuron encoding is prohibitive. Therefore, it is desirable to encode manifolds of stimuli in a more economical way—for example, by representing each point of the manifold by a certain set of neurons. It is an open question how distance relationships between such groups of neurons could be encoded and whether the dynamics from our model could be replicated in such a scenario.

### Embedding into a bigger picture

While the model focuses on the solution of graph traversal problems, it appears desirable to embed it into a broader context of sensory perception, decision making, and motion control in the brain. One particular question is how the hypothetical “puppet string mechanism”—which we postulated to connect proprioception and motion control—could be implemented in a neural substrate. Similarly, if our model provides an appropriate description of place cells and their role in navigation, the question arises how a shift in place cell activity is translated into appropriate muscle commands to propel the animal into the corresponding direction.

It is intriguing to speculate about a deeper connection between our model and object recognition: On the same neural substrate, our hypothetical waves might travel through a space of possible transformations, starting from a perceived stimulus and “searching” for a previously learned representative of the same class of objects. This could explain why recognition of rotated objects is much faster than the corresponding mental rotation task^73^: The former would require only one wave to travel through the cognitive map, while the latter would require many waves to move the bump of activity.

## Conclusion

We have shown that a wide range of cognitive tasks, especially those that involve planning, can be represented as graph problems. To this end, we have detailed one possible role for the recurrent connections that exist throughout the brain as computational substrate for solving graph traversal problems. We showed in which way such problems can be modeled as finding a short path from a start node to some target node in a graph that maps to a manifold representing a relevant task space. Our review of empirical evidence indicates that a theme of connectivity can be observed in the neural structure throughout (at least) the neocortex which is well suited to realize the proposed model.

## Methods and experiments

The model described in the “Proposed model” section above treats the recurrent neural network as a discretized approximation to the manifold of stimuli. Thus, the problem of finding a short path through that manifold translates into a graph traversal problem in the corresponding graph of synaptic connections. In the following, the starting and target position of the planning process are denoted by $s$ and $t$, respectively.

### Neuronal network setup—exemplary implementation of the model

#### Splitting dynamics to two network layers

As described in the “A network of neurons that represents a manifold of stimuli” section, for our numerical implementation of the model, we separated the two different types of dynamics into distinct layers of neurons, the *continuous attractor layer* and the *wave propagation layer*. The split into two layers makes the model more transparent and ensures that parameter changes have limited and traceable effects on the over-all dynamics. As an additional simplification, we do not explicitly model the feed-forward connections which drive the wave propagation layer, but we rather directly activate certain neurons in this layer.

Activation in the *continuous attractor layer* *C* represents the start node $s$, that in the course of the simulation will move towards the target node $t$, which is permanently stimulated in the *wave propagation layer* *P*. Waves of activation are travelling from $t$ across *P*. As soon as the wave front reaches a node in *P* that is connected to a node in proximity to the current activation in *C*, the activation in *C* is moved towards it. Thus, every arriving wave front will pull the activation in *C* closer to $t$, forcing the activation to trace back the wave propagation to its origin $t$.

**Figure 6:**
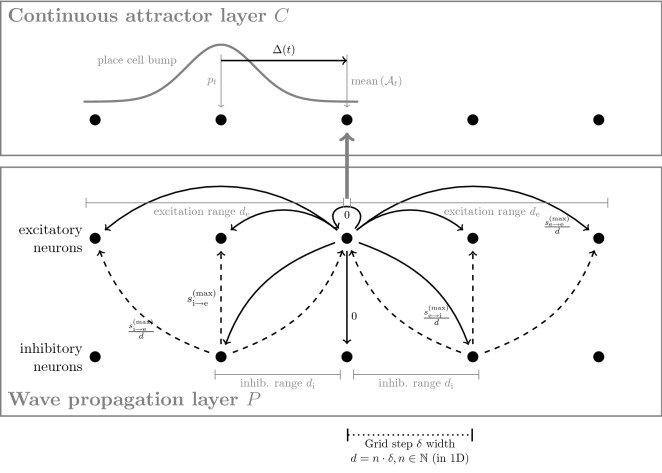
Connectivity of the neurons. For simplicity, this visualization only contains a 1D representation. In the wave propagation layer, excitatory synapses are drawn as solid arrows, dashed arrows indicate inhibitory synapses. Upon its activation, the central excitatory neuron stimulates a ring of inhibitory neurons that in turn suppress circles of excitatory neurons to prevent an avalanche of activation and support a circular wave-like expansion of the activation across the sheet of excitatory neurons. Furthermore, overlap between the active neurons in *C* and *P* is used to compute the direction vector $\Delta (t)$ used for biasing synapses in *C* and thus shifting activity there.

In detail, these dynamics require a very specific network configuration which is described in the following. Figure 6 contains a general overview of the intra- and inter-layer connectivity used in the model and our simulations.

#### Spiking neuron model in the wave propagation layer

In the performed experiments, the wave propagation layer *P* is constructed with an identical number of excitatory and inhibitory Izhikevich neurons^20,21^, that cover a regular quadratic grid of $41\times 41$ points on the manifold of stimuli.

The spiking behavior of each artificial neuron is modeled as a function of its membrane potential dynamics *v*(*t*) using the two coupled ordinary differential equations $\frac{\mathrm {d}}{\mathrm {d}t}v = 0.04 v^2 +5 v + 140 - u + I$ and $\frac{\mathrm {d}}{\mathrm {d}t}u = a\cdot (b v-u)$. Here, *v* is the membrane potential in mV, *u* an internal recovery variable, and *I* represents synaptic or DC input current. The internal parameters *a* (scale of *u* / recovery speed) and *b* (sensitivity of *u* to fluctuations in *v*) are dimensionless. Time *t* is measured in ms. If the membrane potential grows beyond the threshold parameter $v\ge {30}\,mV$, the neuron is spiking and the variables are reset via $v \leftarrow c$ and $u \leftarrow u+d$. Again, *c* (after-spike reset value of *v*) and *d* (after-spike offset value of *u*) are dimensionless internal parameters.

**Table 1:** Parameters used in our simulations of the wave propagation layer *P*.

(a) Neuron model parameters (homogeneous setup)		
	Excitatory	Inhibitory
	RS	FS
*a*	0.02	0.1
*b*	0.2	0.2
*c*	− 65	− 65
*d*	8	2

(b) Neuron model parameters (heterogeneous setup)		
	Excitatory	Inhibitory
	RS ...CH	LTS ...FS
*a*	0.02	$0.02 + 0.08r_i$
*b*	0.2	$0.25 - 0.05r_i$
*c*	$-65 + 15 r_e^2$	− 65
*d*	$8 - 6 r_e^2$	2

(c) Synaptic strength parameters		
$s_\mathrm {e\rightarrow {}e}^\mathrm {(max)}$	50	
$s_\mathrm {e\rightarrow {}i}^\mathrm {(max)}$	0.5${s_\mathrm {e\rightarrow {}e}^\mathrm {(max)}}$	
$s_\mathrm {i\rightarrow {}e}^\mathrm {(max)}$	− 9${s_\mathrm {e\rightarrow {}e}^\mathrm {(max)}}$	
$d_\mathrm {e}$	2	

If not stated otherwise in the following, the parameters listed in Table 1a were used for the Izhikevich neurons in *P*. They correspond to regular spiking (RS) excitatory and fast spiking (FS) inhibitory neurons. In contrast to^20^, neuron properties were not randomized to allow for reproducible analyses. The effect of a more biologically plausible heterogeneous neuron property and synaptic strength distribution is analyzed under Numerical Experiments below. Compared to^20^, the coupling strength in *P* is large to account for the extremely sparse adjacency matrix as every neuron is only connected to its few proximal neighbours in our configuration. Whenever a neuron in *P* is to be stimulated externally, a DC current of $I=25$ is applied to it. As in^20^, the simulation time step was fixed to 1 ms with one sub-step in *P* for numerical stability.

#### Synaptic connections in the wave propagation layer

As depicted in Fig. 6, the excitatory neurons are driving nearby excitatory and inhibitory neurons with a synaptic strength of

1 $$\begin{aligned} s_\mathrm {e\rightarrow {}e}(d)&{:}{=} {\left\{ \begin{array}{ll} \dfrac{s_\mathrm {e\rightarrow {}e}^\mathrm {(max)}}{d}, &{} \text {for } 0 < d\le d_\mathrm {e} \\ 0 , &{} \text {else} \end{array}\right. }, \end{aligned}$$

where $s_\mathrm {e\rightarrow {}i}(d)$ is defined analogously. Here, *d* is the distance between nodes in the manifold of stimuli. For simplicity, we model this manifold as a two-dimensional quadratic mesh with grid spacing $\delta =1$ where some connections might be missing. The choice $s\propto {1}/{d}$ was made to represent the assumption that recurrent coupling will be strongest to nearest neighbours and will decay with distance. Note that (1) in particular implies that we have $s_\mathrm {e\rightarrow {}e}(0),s_\mathrm {e\rightarrow {}i}(0)=0$, which prevents self-excitation. To restrict to only localized interaction, we exclude interaction beyond a predefined excitation range $d_\mathrm {e}$ and inhibition range $d_\mathrm {i}$, respectively. Values of the parameters in the expressions for the synaptic strengths used in the simulations are given in Table 1c.

The inhibitory neurons suppress activation of the excitatory neurons by reducing their input current via synaptic strength

2 $$\begin{aligned} s_\mathrm {i\rightarrow {}e}(d)&{:}{=} {\left\{ \begin{array}{ll} s_\mathrm {i\rightarrow {}e}^\mathrm {(max)}, &{} \text {for } d = 0 \\ \dfrac{s_\mathrm {i\rightarrow {}e}^\mathrm {(max)}}{d}, &{} \text {for } 0 < d\le d_\mathrm {s} \\ 0 , &{} \text {else} \end{array}\right. }\quad . \end{aligned}$$

#### Wave propagation dynamics

**Figure 7:**
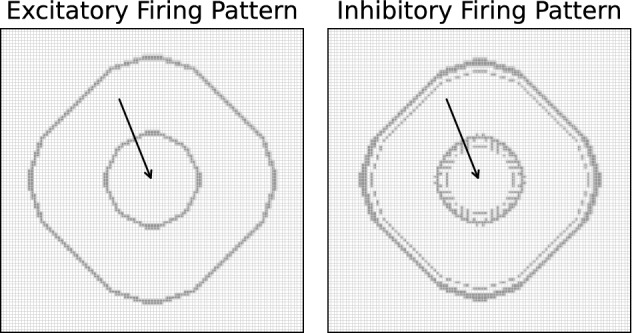
Activity patterns of the excitatory and inhibitory neurons on a $101\times 101$ quadratic neuron grid. Spiking neurons are shown as gray areas. One excitatory neuron at the grid center (arrow) is driven by an external DC current to regular spiking activity. Due to the nearest-neighbour connections, this activity is propagating in patterns that resemble a circular wave structure. The inhibitory neurons prevent catastrophic avalanche-like dynamics by suppressing highly active regions.

The described setup allows for wave-like expansion of neuronal activity from an externally driven excitatory neuron as shown in Fig. 7.

With the capability of propagating signals as circular waves from the target neuron $t$ across the manifold of stimuli in *P*, it is now necessary to set up a representation of the start neuron $s$ in *C*. This will be done in the following subsection before the coupling between *P* and *C* will be described.

#### Neuron model for place cell dynamics

The *continuous attractor layer* *C*, implements a sheet of neurons that models the functionality of a network of place cells in the human hippocampus using rate-coding neurons^17,18^ and thus the manifold of stimuli. As for the wave propagation layer, we also use a quadratic $41\times 41$ grid of neurons for this layer. Activation in the continuous attractor layer will appear as bump, the center of which represents the most likely current location on the manifold of stimuli.

This bump of activation is used to represent the current position in the graph of synaptic connections representing the cognitive map. Planning in the manifold of stimuli thus amounts to moving the bump through the sheet of neurons where each neuron can be thought of as one node in this graph. With respect e. g. to the robot arm example in Fig. 1b, the place cell bump represents the current state of the system i. e. the current angles of the arm’s two degrees-of-freedom. As the bump moves through the continuous attractor layer, and thus through the graph, the robot arm will alter its configuration creating a movement trajectory through the 2D space.

#### Synaptic connectivity to realize continuous attractor dynamics

Our methodology for modelling the continuous attractor place cell dynamics adapts the computational approach used in^19^ by including a computational consideration for synaptic connections between continuous attractor neurons and an associated update rule that depends on information from the wave propagation layer *P*.

The synaptic weight function connecting each neuron in the continuous attractor sheet to each other neuron is given by a weighted Gaussian. This allows for the degrading activation of cells in the immediate neighbourhood of a given neuron and the simultaneous inhibition of neurons that are further away, thus giving rise to the bump-shaped activity in the sheet itself. The mathematical implementation of these synaptic connections also allows for the locus of activation in the sheet to be shifted in a given direction which is, in turn, how the graph implemented by this neuron sheet is able to be traversed.

The synaptic weight $w_{\vec {i},\vec {j}}\in \mathbb {R}^{(N_x\times N_y)\times (N_x\times N_y)}$ connecting a neuron at position $\vec {i}=(i_x, i_y)$ to a neuron at position $\vec {j}=(j_x, j_y)$ is given by

3 $$\begin{aligned} w_{\vec {i},\vec {j}}&{:}{=} J\cdot \exp \left( -\frac{1}{\sigma ^{2}}\left\Vert \left( \frac{i_x-j_x}{N_x},\frac{i_y-j_y}{N_y}\right) +\vec {\Delta }(t)\right\Vert ^{2}\right) -T\,. \end{aligned}$$

Here, *J* determines the strength of the synaptic connections, $\Vert \cdot \Vert$ is the Euclidean norm, $\sigma$ modulates the width of the Gaussian, *T* shifts the Gaussian by a fixed amount, $\vec {\Delta }(t)$ is a direction vector which we discuss in detail later, and $N_{x}$ and $N_{y}$ give the size of the two dimensions of the sheet.

In order to update the activation of the continuous attractor neurons and to subsequently move the bump of activation across the neuron sheet, we compute the activation $A_{\vec {j}}$ of the continuous attractor neuron $\vec {j}$ at time $t+1$ using

4 $$\begin{aligned} B_{\vec {j}}(t+1)&= \sum _{\vec {i}}A_{\vec {i}}(t)w_{\vec {i},\vec {j}}(t)\,, \end{aligned}$$

5 $$\begin{aligned} A_{\vec {j}}(t+1)&= (1-\tau )B_{\vec {j}}(t+1)+\tau \frac{B_{\vec {j}}(t+1)}{\sum _{\vec {i}} A_{\vec {i}}(t)}\,, \end{aligned}$$

where $B_{\vec {j}}(t+1)$ is a transfer function that accumulates the incoming current from all neurons to neuron $\vec {j}$ and $\tau$ is a fixed parameter that determines stabilization towards a floating average activity.

**Table 2:** Parameters for the continuous attractor layer *C*.

$\sigma$	Gaussian width	0.03
*T*	Gaussian shift	0.05
*J*	Synaptic connection strength	12
$\tau$	Stabilization strength	0.8

Simulation parameters for the continuous attractor layer *C* are given in Table 2. They have been manually tuned to ensure development of stable, Gaussian shaped activity with an effective diameter of approximately twelve neurons in *C*.

As in^19^, a direction vector $\vec {\Delta }(t)\in \mathbb {R}^2$ has been introduced in Eq. (3). It has the effect of shifting the synaptic weights in a particular direction which in turn causes the location of the activation bump in the attractor layer to shift to a neighbouring neuron. In other words, it is this direction vector that allows the graph to be traversed by informing the place cell sheet from which direction the wave front is coming in *P*. Thus all that remains for the completion of the necessary computations is to compute $\vec {\Delta }(t)$ as a function of the propagating wave and the continuous attractor position.

#### Layer interaction—direction vector

The interaction between the wave propagation layer *P* and the continuous attractor layer *C* is mediated via the direction vector $\vec {\Delta }(t)$. The direction vector is computed such that it points from the center of the bump of activity towards the center of the overlap between bump and incoming wave as follows. Let $\mathcal {C}_t$ and $\mathcal {P}_t$ denote the sets of positions of active neurons at time *t* in layer *C* and *P*, respectively. Note that each possible position corresponds to exactly one neuron in the wave propagation layer and exactly one neuron in the continuous attractor layer as they have the same spatial resolution in the implementation. Now let $\mathcal {A}_{t}{:}{=} \mathcal {C}_t\cap \mathcal {P}_t$. Then,

6 $$\begin{aligned} \mathop {{\text {mean}}}\left( \mathcal {A}_{t}\right)&= \frac{1}{\left| \mathcal {A}_{t}\right| }\sum _{\vec {i}\in \mathcal {A}_{t}}\vec {i} \end{aligned}$$

is the average position of overlap. We compute the direction vector from the current position $p_t$ of the central neuron in the continuous attractor layer activation bump to $\mathop {{\text {mean}}}\left( \mathcal {A}_{t}\right)$ via

7 $$\begin{aligned} \vec {\Delta }(t)&= \mathop {{\text {mean}}}\left( \mathcal {A}_{t}\right) - p_t\,. \end{aligned}$$

#### Layer interaction—recovery period

In order to prevent the wave from interacting with the back side of the bump in *C* and thus pulling it back again, we introduce a recovery period *R* of a few time steps after moving the bump. During *R*, which is selected as the ratio of bump size to wave propagation speed, $\mathcal {A}_{t}$ is assumed to be empty, which prevents any further movement. In our experiments, we used $R={12}\,ms$. As the bump had a diameter of eleven cells and the maximum wave propagation speed was one cell per ms, this allowed every wave front to interact with the bump at most once.

### Numerical experiments

In order to test the complex neuronal network configuration described in the previous sections and to study its properties and dynamics, we performed numerical experiments using multiple different setups. Source code used for our studies is published at^74^. Results of our simulations are presented in the “Results of the numerical experiments” section. In the following, we will add some more in-depth analyses on specific properties of the model as observed in the simulations.

#### Transmission velocity

In our setup, no synaptic transmission delay, as e. g. in^75^, is implemented. As, due to the strong nearest-neighbour connectivity, only few pre-synaptic spiking neurons are sufficient to raise the membrane potential above threshold, the waves are travelling across *P* with a velocity of approximately one neuronal “ring” per time step, cf. Fig. 4. In contrast, the continuous attractor can only move a distance of at most half its width per incoming wave. Accordingly, its velocity is tightly coupled to the spike frequency of the stimulated neuron while still being bound due to the recovery period *R*.

#### Obstacles and complex setups

In the S-shaped maze Fig. 5a, the continuous attractor activity moves towards the target node $t$ on a direct path around the obstacles. Due to the optimal path being more than two times longer than in Fig. 4, the time to reach the target is accordingly longer as well. This is also in line with the required travel times from $s$ to $t$ in Fig. 5b,c, where—despite its complexity—a path through the maze is found fastest due to it being shorter than in the other cases of Fig. 5. This observation is also evidenced by the fact that our model is a parallelized version of *BFS*, cf. “Relation to existing graph traversal algorithms”, which is guaranteed to find the shortest path in an unweighted and undirected graph.

#### Heterogeneous neuron properties and synaptic strengths

In the simulation experiments described up to now, a homogeneous wave propagation layer *P* is employed. There, all neurons are subject to the same internal parameters, being either regular spiking excitatory neurons or fast spiking inhibitory neurons. Also, synaptic strengths are strictly set as described previously with parameters from Table 1c. This setup is rather artificial. Natural neuronal networks will exhibit a broad variability in neuron properties and in the strength of synaptic connectivity.

To account for this natural variability, we randomized the individual neuron’s internal properties as suggested in^20^, see Table 1b. As in^20^, heterogeneity is achieved by randomizing neuron model parameters using random variables $r_e$ and $r_i$ for each excitatory and inhibitory neuron. These are equally distributed in the interval [0; 1] and vary neuron models between regular spiking ($r_e=0$) and *chattering* (*CH*, $r_e=1$) or fast spiking ($r_i=1$) for excitatory neurons and *low-threshold spiking* (*LTS*, $r_i=0$) for inhibitory neurons. By squaring $r_e$, the excitatory neuron distribution is biased towards RS. In addition, after initializing synaptic strengths in *P*, we randomly varied them individually by up to $\pm {10}\, \%$.

**Figure 8:**
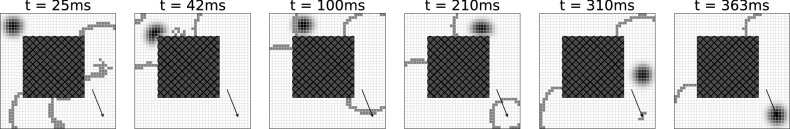
Block setup as in Fig. 5 but with a heterogeneous neuron configuration in *P*.

Despite this strong modification to the original numerically ideal setup, a structured wave propagation is still possible in *P* as can be seen in Fig. 8. While the stereotypical circular form of the wave fronts dissolves in the simulation, they continue to traverse *P* completely. As before, they reach the continuous attractor bump and are able to guide it to their origin. Apparently, the overall connection scheme in *P* is more important for stable wave propagation than homogeneity in the individual synaptic strengths and neuron properties.

An interesting aspect of this simulation when compared to Fig. 5b is the apparent capability of solving the graph traversal problem quicker than with the homogeneous neuronal network. This is an artifact of the explicitly broken symmetry in the heterogeneous configuration: The wave fronts from different directions differ in shape when arriving at the initial position of the continuous attractor layer activity. Thus, one of them is immediately preferred and target-oriented movement of the bump starts earlier than before. This capability of breaking symmetries and thus quickly resolving ambiguous situations is an explicit advantage of the more biologically realistic heterogeneous configuration.

## Supplementary Information

Supplementary Video 1.

Supplementary Video 2.

Supplementary Video 3.

Supplementary Video 4.

Supplementary Video 5.

Supplementary Information.
