# Supplementary material for: A hybrid biological neural network model for solving problems in cognitive planning
Source: Sci Rep. 2022 Jun 23;12:10628. doi: 10.1038/s41598-022-11567-0 (PMC9226121; doi:10.1038/s41598-022-11567-0)
Supplement: Supplementary file 6 — Supplementary Information. [file 41598_2022_11567_MOESM6_ESM.pdf]

Captions for Supplementary Videos

Figure 4 (simple ESM) : Video demonstration of the activity in the wave propagation layer (greyish lines) and the continuous attractor layer (circular blob-like structure) overlaid on top of each other. The grid signifies the neural network structure, i. e. every grid cell in the visualization corresponds to one neuron in each, the wave propagation layer and the continuous attractor layer. The position of the external wave propagation layer stimulation (to-be state) is shown with an arrow. Starting from an initial position in the top left of the sheet, the activation bump traces back the incoming waves to their source in the bottom right

Figure 5 (a,b,c) : Simulations where specific portions of the neural layers were blocked for traversal (dark hatched regions) show the model's capability of solving complex planning problems. Note, that especially in the very fine structure of Fig. 5c leftover excitation can trigger waves apparently spontaneously in the simulation region. As the corresponding neurons are not constantly stimulated, these are usually singular events that do not disturb the overall process.

Figure 8 (central block randomised) : Block setup as in Fig. 5 but with a heterogeneous neuron configuration in P.
